# Engineered probiotics Clostridium butyricum‐pMTL007‐GLP‐1 improves blood pressure via producing GLP‐1 and modulating gut microbiota in spontaneous hypertension rat models

**DOI:** 10.1111/1751-7915.14196

**Published:** 2022-12-18

**Authors:** Xin‐liang Wang, Wen‐jie Chen, Rui Jin, Xuan Xu, Jing Wei, Hong Huang, Yan‐hua Tang, Chang‐wei Zou, Ting‐tao Chen

**Affiliations:** ^1^ Key Laboratory of Poyang Lake Environment and Resource Utilization, School of Resources Environmental and Chemical Engineering Ministry of Education, Nanchang University Nanchang China; ^2^ National Engineering Research Center for Bioengineering Drugs and Technologies Institute of Translational Medicine, Nanchang University Nanchang China; ^3^ Department of Cardiovascular Surgery The Second Affiliated Hospital of Nanchang University Nanchang China

## Abstract

Hypertension is a significant risk factor of cardiovascular diseases (CVDs) with high prevalence worldwide, the current treatment has multiple adverse effects and requires continuous administration. The glucagon‐like peptide‐1 receptor (GLP‐1R) agonists have shown great potential in treating diabetes mellitus, neurodegenerative diseases, obesity and hypertension. Butyric acid is a potential target in treating hypertension. Yet, the application of GLP‐1 analogue and butyric acid in reducing blood pressure and reversing ventricular hypertrophy remains untapped. In this study, we combined the therapeutic capability of GLP‐1 and butyric acid by transforming *Clostridium butyricum* (CB) with recombinant plasmid pMTL007 encoded with hGLP gene to construct the engineered probiotics *Clostridium butyricum*‐pMTL007‐GLP‐1 (CB‐GLP‐1). We used spontaneous hypertensive rat (SHR) models to evaluate the positive effect of this strain in treating hypertension. The results revealed that the intragastric administration of CB‐GLP‐1 had markedly reduced blood pressure and improved cardiac marker ACE2, AT2R, AT1R, ANP, BNP, β‐MHC, α‐SMA and activating AMPK/mTOR/p70S6K/4EBP1 signalling pathway. The high‐throughput sequencing further demonstrated that CB‐GLP‐1 treatments significantly improved the dysbiosis in the SHR rats via downregulating the relative abundance of Porphyromonadaceae at the family level and upregulating *Lactobacillus* at the genus level. Hence, we concluded that the CB‐GLP‐1 greatly improves blood pressure and cardiomegaly by restoring the gut microbiome and reducing ventricular hypertrophy in rat models. This is the first time using engineered CB in treating hypertension, which provides a new idea for the clinical treatment of hypertension.

## Graphical abstract

*Clostridium butyricum*‐pMTL007‐GLP‐1 improved blood pressure, cardiac markers, and dysbiosis in SHR models. The therapeutic effects are mainly contributed to GLP‐1 and butyrate secreted by the engineered probiotics.

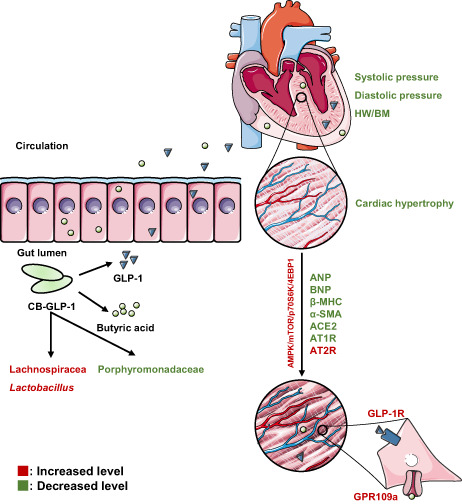

## INTRODUCTION

Hypertension is a serious chronic disease affecting 23.2% of adults in China (Wang et al., 2018) and more than 1.38 billion people worldwide (Mills et al., 2016). The high and ongoing prevalence and medication expenditures of hypertension had been causing a heavy burden to society and patients themselves. The most important clinical feature of hypertension is systolic blood pressure (BP) above 140 mmHg and/or diastolic BP above 90 mmHg (Poulter et al., 2015). The prolonged hypertensive state will lead to functional alteration of a variety of organs and tissues thus, in turn, increasing the risk of multiple diseases (Poulter et al., 2015). Hypertension has been identified as one of the major risk factors for many cardiovascular diseases such as myocardial infarction (MI), coronary heart disease (CHD) and apoplexy (van Oort et al., 2020). Moreover, apart from renal diseases and ocular hypertension, recent studies have also found correlation between hypertension with certain extra‐cardiovascular diseases including Alzheimer's disease (Carnevale et al., 2020; Kivipelto et al., 2018) and diabetes mellitus (Joseph et al., 2021; Zhang, Hou, et al., 2020; Zhang, Nie, et al., 2020).

However, hypertension is a multifactorial disease with vague understanding of its aetiology. The pathogenesis of hypertension is shown to be highly related to lifestyle, genetics, environment and mental state (Poulter et al., 2015). In accordance with the clinical feature and pathophysiology, the treatment of hypertension usually involves balanced diet, appropriate exercise and drug medication (Whelton et al., 2018). Current first‐line antihypertensive drugs are mainly α/β receptor antagonists, angiotensin‐converting enzyme inhibitors (ACEi), angiotensin II receptor blockers (ARBs), calcium channel blockers (CCB), diuretics, direct renin inhibitors and direct vasodilators (Whelton et al., 2018). Nevertheless, the aforementioned drugs require long‐term administration and have many adverse effects (Tsioufis & Thomopoulos, 2017).

Many studies indicated the significance of intestinal microecology in regulating BP (Lynch & Pedersen, 2016). Improvement of cardiovascular function mediated by gut microbiota is mainly due to neuro‐regulation via brain‐gut axis, immunoregulation and bioactive metabolite (Avery et al., 2021; Santisteban et al., 2016; Touyz & Camargo, 2019). Most notably, butyric acid, a representative of short‐chain fatty acids (SCFAs), is a bioactive metabolite generated by beneficial bacteria and is highly active in regulating BP, dilating vessels and reducing inflammatory reactions (Avery et al., 2021). Recent studies have shown that BP normalisation induced by change of lifestyle is attributed to elevated butyric acid from intestinal microecological restoration (Maifeld et al., 2021; Touyz, 2021). The microbial alteration of hypertension patients is manifested by a reduction of *Akkermansia* strains as well as overgrowth of *Prevotella* and *Klebsiella* strains at the genus level, which leads to marked reduction of serum butyric acid (Li et al., 2017; Naqvi et al., 2021). Another research about Australian people had been demonstrated that hypertensive patients were associated with increased *Clostridium* and *Prevotella* while normal people with lower BP have more *Alistipesfinegoldii* and *Lactobacillus* at genus level (Dinakis et al., 2022). It is suggested that butyrate has much stronger therapeutic effect than other SCFAs (Muralitharan et al., 2020). *Clostridium butyricum* (CB) is a probiotic bacterium with great ability to pass gastrointestinal tract and secret butyric acid, which has been preclinically applied to treat obstructive sleep apnoea‐associated hypertension (Ganesh et al., 2018). Yet, the direct effect of CB on hypertension remained unclear.

Glucagon‐like peptide‐1 (GLP‐1), a low half‐life molecule secreted by enteral L cells, has been cultivating its anti‐diabetic and weight‐reducing function (Drucker, 2018). Furthermore, studies have pointed out the potential of GLP‐1 in improving BP (Li et al., 2017). Currently, GLP‐1 receptor (GLP‐1R) agonists and dipeptidyl peptidase‐4 inhibitors (DPP‐4i) are approved clinically. However, GLP‐1 analogues such as liraglutide, exenatide, exenatide microspheres and duratide require periodic injection, leading to low compliance. In addition, DPP‐4i may lead to severe pancreatitis and certain types of DPP‐4i could cause cardiac and hepatic deterioration (Lee et al., 2019; Scheen, 2018; Sinha & Ghosal, 2019). Thus, developing highly compliant GLP‐1‐targeted drugs with minor adverse effects is demanded. In our previous studies, we presented the potency of using GLP‐1‐expressing engineered bacteria in the treatment of multiple diseases (Chen et al., 2018; Fang et al., 2019, 2020; Luo et al., 2021; Wang et al., 2021; Wu et al., 2022). Whereas we did not fully investigate the possible role of butyrate and neither single use of GLP‐1 nor the combination of two candidate antihypertensive substances butyric acid and GLP‐1 was investigated in cardiovascular diseases.

Herein, we constructed engineered bacteria *Clostridium butyricum*‐GLP‐1 that is capable of secreting GLP‐1 and butyric acid to treat spontaneous hypertensive rats (SHR) via oral administration of probiotic suspension and assess the cardiac improvement effect of the engineered probiotic strain on hypertension. The regulatory effectiveness of the engineered probiotic strain is quantified by sphygmomanometer, western blotting and histopathology. The restoration of dysbacteriosis is determined by 16S ribosomal DNA (rDNA) high‐throughput sequencing.

## EXPERIMENTAL PROCEDURES

### Construction and evaluation of the engineered bacteria in vitro

The recombinant plasmid pMTL007‐GLP‐1 was synthesised by Suzhou GENEWIZ Co., Ltd. by integrating the hGLP‐EGFP gene into the 5′ HindIII to 3′ BsrGI site of pMTL007 plasmid (GenBank: EF525477.1), a specialised plasmid for *Clostridium* (Heap et al., 2007). Then, the receptor bacteria CB was transformed with the recombinant plasmid pMTL007‐GLP‐1 by heat shock. The growth curves, plasmid stability, acid resistance, bile salt resistance and anti‐oxidative capability were evaluated.

### Experimental design and treatment of animals

The experimental animals include 24 spontaneously hypertensive rats (SHR) and six Wistar rats were all aged 8 weeks, purchased from Beijing Vital River Laboratory Animal Technology Co., Ltd. and fed freely with standardised diet and water under consolidated conditions (humidity 50 ± 15%, temperature 22 ± 2°C, 12/12 light–dark cycle) in the specific pathogen‐free (SPF) experimental animal barrier system of the Institute of Translational Medicine of Nanchang University (Nanchang, Jiangxi Province, China). After adaptation for 1 week, the experimental animals are randomly divided into five groups: (1) C group, a control group that contains six Wistar rats; (2) M group, a model group that contains six SHR rats; (3) CB group, a group that contains six SHR rats treated with 10^9^ CFU/ml CB via gavage every 2 days until the end of the treatment; (4) CB‐GLP‐1 group that contains six SHR rats treated with 10^9^ CFU/ml CB‐GLP‐1 via gavage every 2 days until the end of the treatment; and (5) EX group, a group that contains six SHR rats treated with 0.4 mg/kg exenatide injected intraperitoneally every 2 days until the end of the treatment. The rats were euthanised by qualified laboratory technicians by injecting 1% sodium pentobarbital (40 mg/kg) intraperitoneally following dissection at the end of the terminal treatment session for later experiments. The flow chart of the experimental animals' treatment is shown in Figure 2A.

### Blood pressure tests

The initial systolic and diastolic BP of all rats were measured by Mouse and Rat Tail Cuff Blood Pressure Systems from IITC Life Science Inc. following instructions from the provider. During the treatment session, the blood pressure of each rat was measured weekly. To reduce experimental errors, the BP was measured every Monday and the average value is obtained by measuring three times in each rat until the end of the experiment.

### Histology and histopathology

The euthanised rat was immediately dissected, and the heart was selected at random. Then, after fixation using 4% paraformaldehyde, the heart tissues were embedded in paraffin. Next, the heart tissues embedded in paraffin were sectioned by microtome into 2–4 μm serial cuts, following dewaxing, staining with haematoxylin and eosin (H&E) or Masson, dehydration and clearing. The stained section of the heart tissues is then sealed with Permount for observation under the light microscope.

### Western blotting

Take an appropriate amount of heart tissue into the centrifuge tube and add an appropriate amount of RIPA lysis buffer (Beijing Solarbio Science & Technology Co., Ltd.) and protease inhibitor. After tissue homogenate treatment on ice, the supernatant was collected by centrifugation at 12,000 *g* at 4°C for 10 min, and the protein concentration was measured. Then, the cell protein was isolated by 10%–12% gel electrophoresis (SDS‐PAGE) and transferred to a polyvinylidene fluoride (PVDF) membrane. At room temperature, 5% non‐fat milk dissolved in Tris buffer saline Tween (TBST) was used to block the non‐specific binding site for 1 h. The PVDF membrane was then incubated with appropriately diluted primary antibody at 4 °C overnight (Table S1), and after washing with TBST, the secondary antibody diluted with 1% dry milk TBST was incubated at room temperature for 60 min. The western blotting results were obtained by adding the proper amount of enhanced chemiluminescence to the PVDF membrane and exposing on an automatic gel imaging system.

### High‐throughput sequencing of 16S rDNA amplicon

The bacterial genomic DNA amplifying and sequencing was performed by Realbio Technology (RBT) Co., Ltd. The target of the fragment of the 16S subunit of bacterial ribosomal RNA V3‐V4 region in faecal samples was amplified using bacterial universal primers 341F (5′‐CCTACGGGRSGCAGCAG‐3′) and 802R (5′‐GGACTACVVGGGTATCTAATC‐3′) containing index and adapter sequence. The amplified products of 425 bp (excluding index and adapter sequence) were harvested to compile library. After library quality inspection with agarose gel electrophoresis, library quantification was performed using Qubit and proportionally mixed according to the amount of data required for each sample. Next, the colony DNA fragments of the amplified products were sequenced using the Ilumina NovaSeq PE250 platform.

### Data analysis

High‐throughput sequencing analysis including chimerism and cluster analysis was performed by Usearch software platform. During Usearch clustering, reads were first sorted according to their abundance from large to small, and then clustered by 97% similarity to obtain operational taxonomic units (OTUs). The α Diversity (OTU, Chao1, Shannon, Simpson and Alpha Diversity indices) and β diversity (Anosim analysis, MRPP analysis, PCoA and NMDS) were analysed. Statistical analysis was performed using GraphPad Prism8.0 (Graph Pad Software). Numerical data are presented as means ± standard deviation (SD). Statistical significance was evaluated by one‐way ANOVA (and nonparametric or mixed) followed by Tukey's multiple comparison tests. Statistical significance was set at *p < 0.05, **p < 0.01, ***p < 0.001 and ****p < 0.0001.

## RESULTS

### Evaluation of probiotic characteristics of CB‐GLP‐1 in vitro

**FIGURE 1:**
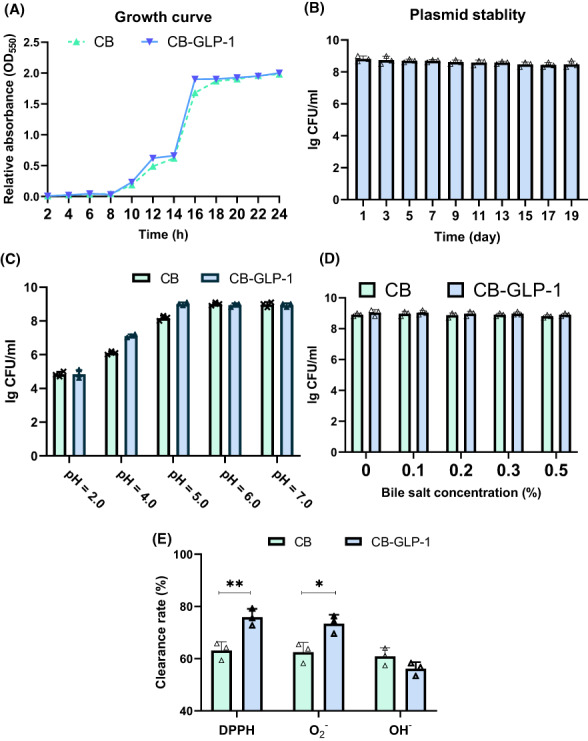
Evaluation of the probiotic characteristics of CB‐GLP‐1. Values are presented as means ± SD (*n* = 3); (A) Growth curves of wild‐type CB and engineered stain CB‐GLP‐1; (B) Plasmid stability test of CB‐GLP‐1; (C) The acid tolerance of CB and CB‐GLP‐1; (D) The bile salt tolerance of CB‐GLP‐1; (E) The antioxidant capability of CB‐GLP‐1; *p < 0.05 and **p < 0.01.

The growth curve assay has indicated no difference in growth characteristics between CB and CB‐GLP‐1 strains (Figure 1A). The plasmid stability of CB‐GLP‐1 was then assessed and suggested that viable CB‐GLP‐1 still reached 5 × 10^8^ CFU/ml after 19 days of passaging once per day (Figure 1B). In addition, the resistance of CB and CB‐GLP‐1 to high concentrations of acid and bile salts was evaluated, respectively, and showed that both CB and CB‐GLP‐1 showed great resistance to acid and bile salts (Figure 1C,D), which suggested that both CB‐GLP‐1 and CB had the ability to resist and survive the gastrointestinal environment. Finally, the antioxidative properties were evaluated, both CB and CB‐GLP‐1 showed good antioxidative capacity, especially in 2,2‐diphenyl‐1‐picrylhydrazyl (DPPH) radical and $O_{2}^{-}O$ reducing capacity, with CB‐GLP‐1 scavenging capacity being superior to CB (Figure 1E, *p* < 0.05).

### Engineered bacteria ameliorated blood pressure and decreased ventricular hypertrophy in rat models

**FIGURE 2:**
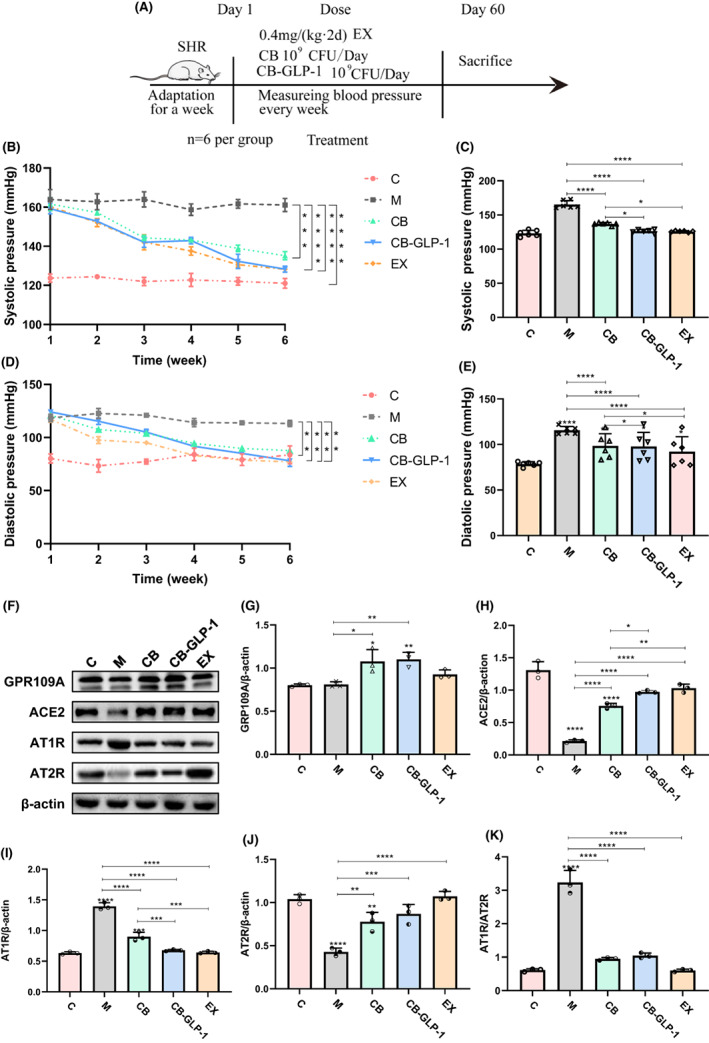
CB‐GLP‐1 treatment reduces blood pressure in rats. Values are presented as means ± SD (*n* = 3); (A) A schedule of animal experiments; (B) Systolic pressure of rats; (C) Systolic pressure of rats at the end of treatment session; (D) Diastolic pressure of rats; (E) Diastolic pressure of rats at the end of treatment session; (F) Western blotting of GPR109A, ACE2, AT1R and AT2R expression in heart tissues, the relative expressions of the detected protein were quantified by ImageJ. β‐actin was used as an internal control. The activity of (G) GPR109A, (H) ACE2, (I) AT1R, (J) AT2R and (K) AT1R/AT2R, *p < 0.05, **p < 0.01, ***p < 0.001 and ****p < 0.0001.

In order to observe the effects of CB and engineered bacteria CB‐GLP‐1 on blood pressure in SHR rats, we monitored the changes in blood pressure after probiotics treatment (Figure 2B–E). It was found that compared with the SHR group, systolic and diastolic blood pressure decreased significantly 6 weeks after bacterial and exenatide intervention (Figure 2C,E, *p* < 0.05). After that, we detected the expression of related proteins in the renin‐angiotensin‐aldosterone (RAAS) system by western blotting at the molecular level (Figure 2F). It was found that with the GLP‐1 intervention, the level of angiotensin II (detected by ELISA, data not shown) and the expression of angiotensin II type‐1 receptor (AT1R) were decreased, while the expression of angiotensin II type‐2 receptor (AT2R) and angiotensin‐converting enzyme 2 (ACE2) was increased (Figure 2H–J). We also found that the therapeutic effect mediated by butyric acid depended on increasing the expression level of G‐protein‐coupled receptor 109 A (GPR109A) (Figure 2G). Therefore, we conclude that GLP‐1 and butyric acid have the effect of treating the blood pressure in spontaneously hypertensive rats.

**FIGURE 3:**
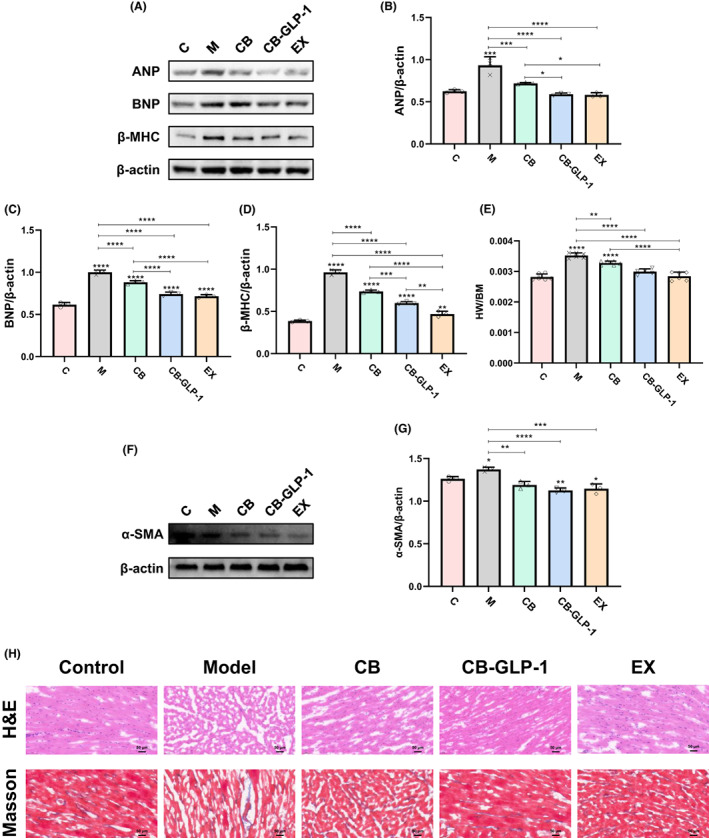
CB‐GLP‐1 reduced cardiac hypertrophy. Values are presented as means ± SD (*n* = 3). (A) Western blotting of ANP, BNP and β‐MHC expression in heart tissues. The relative expressions of (B) ANP, (C) BNP and (D) β‐MHC were quantified by ImageJ, and β‐actin was used as an internal control; (E) Heart weight to body mass ratio (HW/BM) of rats; (F) Western blotting of α‐SMA expression in heart tissues; (G) The relative expressions of α‐SMA quantified by ImageJ, and β‐Actin was used as an internal control; (H) H&E and Masson staining image of heart tissue (400×), *p < 0.05, **p < 0.01, ***p < 0.001 and ****p < 0.0001.

Next, to study the effect of GLP‐1 on myocardial hypertrophy, we intervened in hypertensive rats with bacteria and exenatide for 6 weeks and observed the changes before and after the intervention at the molecular and histological levels (Figure 3). At the molecular level, we detected the expression of cardiac hypertrophy markers by western blotting (Figure 3A). Compared with the control group, the levels of cardiac hypertrophy markers including atrial natriuretic peptide (ANP), brain/B‐type natriuretic peptide (BNP) and β‐myosin heavy chain (β‐MHC) increased significantly (*p* < 0.05) in CB‐GLP‐1 group, while ANP and BNP decreased markedly after GLP‐1 treatment, β‐MHC levels (*p* < 0.05) (Figure 3B–E). At the histological level, the cross‐section of cardiomyocytes in the SHR group increased and improved after bacterial administration and exenatide intervention compared with the control group in H&E staining (Figure 3H). The Masson staining is used to evaluate the deposition of collagen, and a large amount of collagen deposition between the myocardium and around the blood vessels in the SHR group was found, which was improved after the intervention (Figure 3H). These changes were consistent with the expression of markers of myocardial hypertrophy. Interestingly, we found merely slight changes in α‐smooth muscle actin (α‐SMA) among groups (Figure 3F,G).

### CB‐GLP‐1 exerts antihypertensive effects via activating AMPK/mTOR/p70S6K/4EBP1 signalling pathway in rats

**FIGURE 4:**
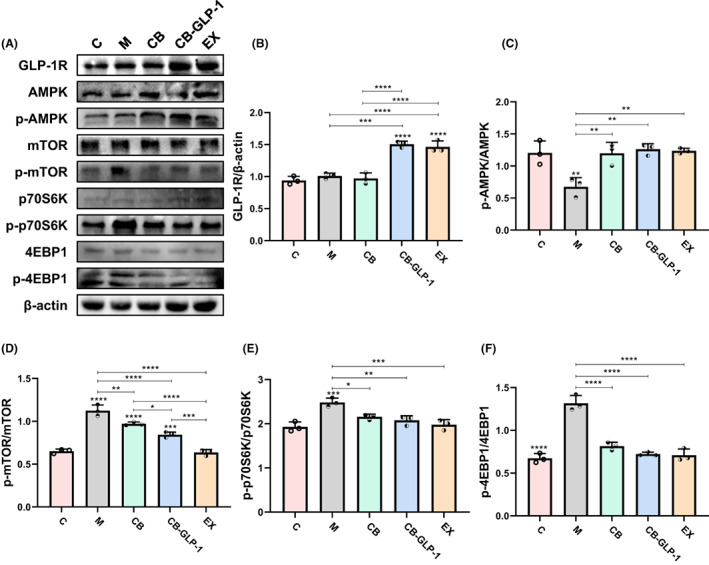
CB‐GLP‐1 exerts antihypertensive effects via activating the AMPK/mTOR/p70S6K/4EBP1 signalling pathway in rats. Values are presented as means ± SD (*n* = 3). (A) Western blotting of GLP‐1R, AMPK, p‐AMPK, mTOR, p‐mTOR, 70S6K, p‐70S6K, 4EBP1 and p‐4EBP1 expression in heart tissues. The relative expressions of (B) GLP‐1R, (C) p‐AMPK/AMPK, (D) p‐mTOR/mTOR, (E) p‐70S6K/70S6K and (F) p‐4EBP1/4EBP1 were quantified by ImageJ, β‐actin was used as an internal control, *p < 0.05, **p < 0.01, ***p < 0.001 and ****p < 0.0001.

It has been found that AMP‐activated protein kinase (AMPK) plays an important role in regulating cell proliferation and apoptosis, and activation of AMPK can inhibit cardiac hypertrophy (Gélinas et al., 2018), and GLP‐1 can inhibit vascular smooth muscle cell proliferation through activation of AMPK (Jojima et al., 2017). Thus, we are interested in whether CB‐GLP‐1 exerts an antihypertensive effect via activating the AMPK‐associated pathway. The western blotting confirmed that the engineered anaerobe CB‐GLP‐1 activated of AMPK/mTOR/p70S6K/4EBP1 signalling pathway in rats (Figure 4). The activation of AMPK inhibits mTOR/p70S6K/4EBP1. Activation of mTOR promotes phosphorylation of eukaryotic translation initiation factor 4E‐binding protein 1 (4EBP1) and ribosomal 40S subunit S6 protein kinase (p70S6K), which are involved in protein translation initiation and elongation (Morita et al., 2015). Studies have shown that the mechanistic target of the ribosomal protein 70 S6 kinase (p70S6K) pathway is involved in stimulating protein synthesis and regulating cardiac hypertrophy (Heineke & Molkentin, 2006). Exenatide, as a GLP‐1R agonist, can regulate cell proliferation and apoptosis seems also by activating AMPK/mTOR/p70S6K/4EBP1 signalling pathway (Zhou et al., 2015). It can also regulate myocardial hypertrophy through this pathway.

### Effect of engineered bacteria treatment on the gut microbiota of hypertension rat models

**FIGURE 5:**
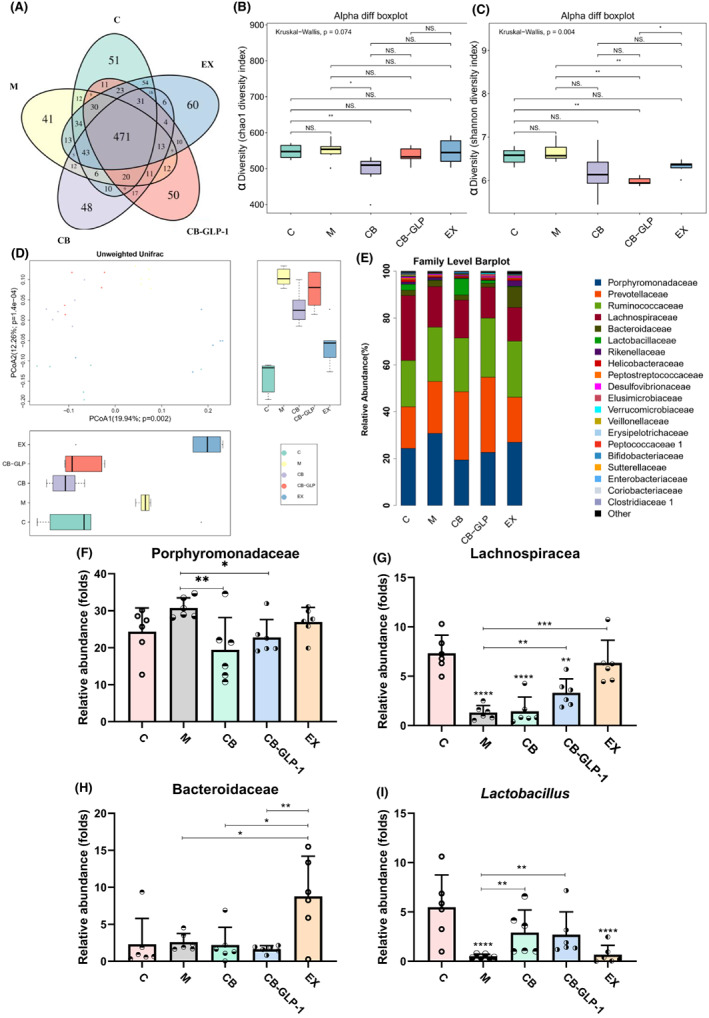
CB‐GLP‐1 improved intestinal microbiota in SHR rats. Values are presented as means ± SD (*n* = 5). (A) Venn map representation of OTUs; (B) The observed species diversity index; (C) The Shannon diversity index; (D) PCoA of β diversity index; (E) Microbial composition at the family level; The relative abundance of (F) Porphyromonadaceae, (G) Lachnospiraceae, (H) Bacteroidaceae and (I) *Lactobacillus* were analysed, ns: no significance, *p < 0.05, **p < 0.01, ***p < 0.001 and ****p < 0.0001.

The alteration of intestinal microbiota of SHR rats was determined by high‐throughput 16S rDNA. A total of 29 samples generated a total of 1144 OTUs, and 471 common OTUs were identified from all groups using the Venn method (Figure 5A). The unique OTUs numbers in C, M, EX, CB and CB‐GLP‐1 were 51, 41, 60, 48 and 50 respectively (Figure 5A). To better analyse the effect of CB‐GLP‐1 intervention on intestinal microbiota in the group CB‐GLP‐1, α diversity analysis of Observed index (used for species diversity) and Shannon index (used for community diversity) was performed (Figure 5B–D).

Our results showed that no obvious changes were identified between group C and group M. While compared with group C, group CB and CB‐GLP‐1 have reduced microbial diversity and abundance (Figure 5E). PCoA analysis showed that dots aggregated in the M group and dispersed in the C group and EX group, while CB‐GLP‐1 treatment promoted the aggregation of dots (Figure 5B–D). In addition, the samples in CB and CB‐GLP‐1 groups showed close similarity, which was far different from that in the C group, which meant that the microbial diversity in CB and CB‐GLP‐1 groups was substantially different from that in the C group (Figure 5B–D).

At the family level, the first 20 microbial populations were analysed. Porphyromonadaceae, Prevotellaceae, Ruminococcaceae and Bacteroidaceae constitute the common dominant bacteria in group C (0.243 vs. 0.178 vs. 0.198 vs. 0.023), group M (0.307 vs. 0.221 vs. 0.232 vs. 0.026), group CB (0.194 vs. 0.291 vs. 0.229 vs. 0.021), group CB‐GLP‐1 (0.226 vs. 0.321 vs. 0.253 vs. 0.016) and group EX (0.269 vs. 0.193 vs. 0.239 vs. 0.087) (Figure 5E). Moreover, CB and CB‐GLP‐1 treatments immensely decreased the relative abundance of Porphyromonadaceae, while there was no significant difference in the relative abundance of Prevotellaceae and Ruminococcaceae between groups, and the abundance of Bacteroidaceae was not quite different from group C after the intervention of CB and CB‐GLP‐1 (Figure 5E).

## DISCUSSION

Hypertension is one of the world's major health problems, defined as systolic blood pressure above 140 mmHg or diastolic blood pressure above 90 mmHg (Mills et al., 2016). Hypertension affects 25% of the general population and is the number one risk factor for serious diseases affecting the brain, heart and kidneys (Messerli et al., 2007). Most hypertensive patients need to take three or more antihypertensive drugs continuously, and most of them are still hypertensive because they cannot be cured (Carey et al., 2018). However, long‐term hypertension can cause pressure on the blood vessel wall, increase the thickness of the blood vessel wall and decrease blood flow, so that the endothelial cells of the blood vessel will be damaged, and the smooth muscle of the blood vessel will also start to proliferate, and the heart will also cause cardiac hypertrophy and diastolic dysfunction, which may eventually lead to heart failure (Poulter et al., 2015). GLP‐1 is a peptide produced by distal intestinal mucosal endocrine cells, apart from its effect on glucose metabolism, GLP‐1 has anti‐inflammatory and antioxidant effects in cell types associated with atherosclerosis formation, as well as direct cardioprotective effects (Krasner et al., 2014; Shiraki et al., 2012). The widespread distribution of GLP‐1 receptors in the human body underlies the role of GLP‐1 in organs other than the pancreas. So far, there has been evidence that GLP‐1 can effectively reduce systolic and diastolic blood pressure, and has attracted much attention for its cardioprotective effect (Wang et al., 2013). However, the therapeutic mechanism of GLP‐1 on hypertension and its inducing cardiac hypertrophy has not been fully explained. Therefore, studying the effect of GLP‐1 on the treatment of hypertension and clarifying the therapeutic value of GLP‐1 in hypertension‐related diseases can provide a new treatment idea for the treatment of hypertension.

In this study, we proposed the treatment of hypertension with engineered bacteria and conducted several experiments to verify its therapeutic function and explore its potential mechanism. In vitro growth curve test, plasmid stability test and GLP‐1 protein expression test showed that CB‐GLP‐1 was successfully constructed and had good plasmid stability and GLP‐1 protein expression ability. Acid tolerance and bile salt tolerance test showed that CB‐GLP‐1 had the ability to tolerate gastric acid and oral potential. The following experimental results showed that there was no significant difference between CB‐GLP‐1 and CB in the scavenging ability of OH^−^ radical and $O_{2}^{-}$ radical, and there was no significant difference between CB and CB‐GLP‐1 in the antioxidant ability (Figure 1). Next, we used the spontaneous hypertensive rat (SHR) models to explore the antihypertensive effect of CB‐GLP‐1 on hypertension. Two to five weeks after CB‐GLP‐1 intervention, the systolic and diastolic blood pressure of SHR was significantly decreased, with the reduction of systolic blood pressure more obvious (Figure 2B–E). The result may attribute to GLP‐1, expressed by CB‐GLP‐1, binding to GLP‐1R in the kidney and then regulating the RAAS system. In this study, we found that the expression of angiotensin II (detected by ELISA, data not shown) and AT1R increased in SHR rats, while the expression of AT2R decreased, resulting in increased secretion of angiotensin II (Figure 2I–K). These results were reversed 6 weeks after the drug intervention. Previous studies have shown that angiotensin II binds to its receptors to contract blood vessels, raise blood pressure and promote cell hypertrophy, which plays an important role in myocardial remodelling and the development of hypertension (Banks et al., 2022). In addition, we also found that with the down‐regulation of AT1R, the expression of ACE2 increased significantly (Figure 2H,I). Therefore, we believed that GLP‐1 could activate ACE2 by inhibiting angiotensin II synthesis and the expression of its receptor, thus playing a role in lowering blood pressure. These results were consistent with the conclusions of the previous studies (Morrell & Stenmark, 2013). Some studies have shown that butyrate regulates the occurrence and development of hypertension through immune response, and also induces the differentiation of regulatory T cells in vivo. When butyrate was applied to its receptor GPR109A, macrophages showed a higher ability to induce juvenile cells to differentiate into regulatory T cells. In our study, the level of receptor GPR109A was substantially increased after CB‐GLP‐1 intervention (Figure 2G). These results suggest that the antihypertensive effect of CB‐GLP‐1 may be partly derived from the secretion of butyric acid in the treatment of hypertension by activating receptor GPR109A.

Many studies have shown that hypertension can produce significant hemodynamic changes, increasing vascular inflammation and myocardial load, inducing cardiac hypertrophy and causing cardiac dysfunction. However, these anti‐inflammatory and cardiac protective effects have been observed in natural GLP‐1 or GLP‐1R agonists (Marso et al., 2016). Therefore, the improvement effect of engineering bacterium CB‐GLP‐1 on myocardial hypertrophy in spontaneous hypertension was investigated in this study (Figure 3). Western blotting was used to detect the levels of myocardial hypertrophy markers ANP, BNP and β‐MHC, and the results showed that the expression levels of myocardial hypertrophy markers were significantly increased in the SHR model (Figure 3B–D). After CB, CB‐GLP‐1 and EX intervention, the expression levels of ANP, BNP and β‐MHC were all decreased (Figure 3B–D). And the effect of CB‐GLP‐1 and EX is better than CB (Figure 3B–D). AMPK, as an energy sensor and regulator of energy homeostasis in eukaryotic cells, is activated when AMP/ATP and ADP/ATP ratios increase in the body, restoring energy balance by inhibiting anabolic processes that consume ATP and promoting catabolic processes that produce ATP (Garcia & Shaw, 2017). It has been found that AMPK also plays an important role in regulating cell proliferation and apoptosis, and activation of AMPK can inhibit cardiac hypertrophy (Gélinas et al., 2018), and GLP‐1 can inhibit vascular smooth muscle cell proliferation through activation of AMPK (Jojima et al., 2017). Activation of AMPK inhibits various anabolic pathways, including mTOR/p70S6K/4EBP1. Activation of mTOR promotes phosphorylation of eukaryotic translation initiation factor 4E‐binding protein 1 (4EBP1) and ribosomal 40S subunit S6 protein kinase (p70S6K), which are involved in protein translation initiation and elongation (Morita et al., 2015). Studies have shown that the mammalian target (mTOR)/P70 ribosomal S6 protein kinase (p70S6K) pathway is involved in stimulating protein synthesis and regulating cardiac hypertrophy (Heineke & Molkentin, 2006). Exenatide, as a GLP‐1R agonist, can regulate cell proliferation and apoptosis by activating AMPK/mTOR/p70S6K/4EBP1 signalling pathway. It can also regulate myocardial hypertrophy through this pathway. Therefore, to confirm our hypothesis, we detected the expression of signalling pathway‐related proteins. In this study, western blotting was used to detect the phosphorylation levels of AMPK, mTOR, p70S6K and 4EBP1 (Figure 4). After the intervention of engineered bacteria and exenatide, AMPK was activated, and mTOR while phosphorylation levels of p70S6K and 4EBP1 were reduced, suggesting that GLP‐1 may be involved in the treatment of hypertensive hypertrophy through activation of AMPK/mTOR/p70S6K/4EBP1 signalling pathway (Figure 4).

Due to the rise of 16S rDNA technology, an increasing number of studies have shown a strong link between gut microbiome and hypertension. Shannon's index in alpha diversity and PCoA results in beta diversity suggest that CB‐GLP‐1 appears to promote the conversion of the SHR gut microbiota to normal control rats (Figure 5). The results at the top 10 family‐level and genus‐level species composition indicated that CB‐GLP‐1 intervention had an inhibitory effect on the Porphyromonadaceae and enhance the relative abundance of probiotic bacteria, including the *Lactobacillus* and Lachnospiraceae (Figure 5). Among them, *Lactobacillus* has the effect of degrading sugar substances to regulate intestinal function, enhance immunity and exert antioxidation ability (Zhang, Hou, et al., 2020; Zhang, Nie, et al., 2020). In addition, the family Lachnospiraceae belongs to the Clostridium cluster XIVa of Firmicutes, and many studies have shown that Lachnospiraceae is the main producer of short‐chain fatty acid in the human intestine mediated by hydrolyses starch and other sugars to produce butyrate, propionate and other short‐chain fatty acids to improve intestinal inflammation, and provides energy to the intestinal epithelium, maintains an acidic environment in the intestine and inhibits the growth of harmful acid intolerant flora, while acid‐tolerant *Lactobacillus*, *Clostridium* and other beneficial bacteria are able to proliferate (Chen et al., 2017). The increase in the abundance of beneficial bacteria in the host gut microbiota is accompanied by an increase in the content of SCFAs, which are coupled to butyric acid secreted by CB‐GLP‐1, thereby activating the SCFAs receptor GPR109A in the kidney to reduce hypertension (Felizardo et al., 2019). It was found that SCFAs and their metabolites interacted with the RAAS system in the host kidney and downregulated RAAS in the kidney of experimental rat under the influence of a high‐fibre diet, greatly downregulating systolic and diastolic blood pressure (Marques et al., 2017). In addition, the intervention of CB‐GLP‐1 improved the gut microbiota disorder caused by hypertension, suggesting that CB‐GLP‐1 may regulate the gut microbiota through the probiotic properties of the host bacterium CB, further suggesting that another mechanism of CB‐GLP‐1 treatment of hypertension is the regulation of the gut microbiota through the host bacterium CB (Figure 5).

**FIGURE 6:**
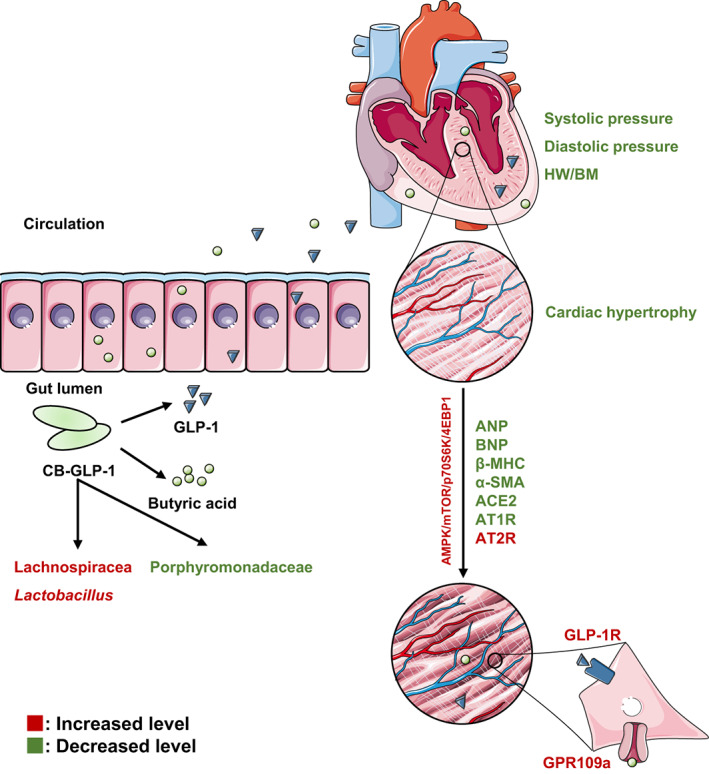
The underlying mechanism of therapeutic effect of CB‐GLP‐1.

Collectively, this study shows that CB‐GLP‐1 treatment of hypertension is mediated by the expression of GLP‐1 and secretion of butyric acid to regulate the RAAS system and GPR109A in the kidney and that CB‐GLP‐1 activates the AMPK signalling pathway to regulate myocardial proliferation and apoptosis and ameliorate cardiomyocyte hypertrophy and ventricular wall fibrosis, in addition, CB‐GLP‐1 treatment through the host bacterial CB increased the abundance and biodiversity of probiotic bacteria, transforming the hypertension‐affected microbial ecology to normal levels (Figure 6). This experiment is the first to investigate the potential role of the engineered bacterium CB‐GLP‐1 in regulating hypertension as well as ameliorating myocardial hypertrophy.

In conclusion, our results showed that genetically engineered bacteria CB‐GLP‐1 have great antihypertensive potential in treating SHR hypertension rat models (Figure 6). The plausible mechanism of the BP‐reducing ability of the CB‐GLP‐1 strain may be attributed to elevating short‐chain fatty acid levels in blood and increasing intestinal microbial diversity of the hypertensive rat. Our findings suggest that the genetically engineered bacteria expressed GLP‐1 and short‐chain fatty acids are putative therapeutics for the treatment of hypertension. Nevertheless, the miss of vector‐carrying strain and the limited number of hypertension rats used in our present study is a non‐negligible influence for us to deduce a better statistical conclusion.

## AUTHOR CONTRIBUTIONS

**Xin‐liang Wang:** Conceptualization (equal); data curation (lead); formal analysis (lead); investigation (lead); methodology (lead); software (lead); validation (equal); visualization (lead); writing – original draft (equal); writing – review and editing (supporting). **Wen‐jie Chen:** Investigation (supporting); methodology (supporting); software (supporting); validation (supporting); visualization (supporting); writing – original draft (equal); writing – review and editing (equal). **Rui Jin:** Investigation (supporting); methodology (supporting); validation (supporting). **Xuan Xu:** Investigation (supporting); methodology (supporting); validation (supporting). **Jing Wei:** Investigation (supporting); supervision (supporting). **Hong Huang:** Funding acquisition (supporting); supervision (supporting); validation (supporting). **Yan‐hua Tang:** Supervision (supporting); validation (supporting). **Chang‐wei Zou:** Supervision (equal); validation (equal). **Ting‐tao Chen:** Conceptualization (lead); funding acquisition (lead); supervision (lead); validation (lead); writing – review and editing (lead).

## FUNDING INFORMATION

This work was supported by grants from the National Natural Science Foundation of China (No. 82060638 to T. T. Chen and No. 42265011 to H. Huang) and Double thousand plan of Jiangxi Province (High End Talents Project of Scientific and Technological Innovation to T. T. Chen).

## CONFLICT OF INTEREST

The authors declare that there is no conflict of interest regarding the publication of this paper.

## ETHICS STATEMENT

The ethical investigation of the experimental animals in our study was approved by the animal experimental ethical inspection of Nanchang Royo Biotech Co., Ltd (RyE2021070911) on August 2 2022.

## Supporting information


Table S1

## Data Availability

The datasets used and/or analysed during the present study are available from the corresponding author on reasonable request.
