# Supplementary material for: Engineered probiotics Clostridium butyricum‐pMTL007‐GLP‐1 improves blood pressure via producing GLP‐1 and modulating gut microbiota in spontaneous hypertension rat models
Source: Microb Biotechnol. 2022 Dec 18;16(4):799–812. doi: 10.1111/1751-7915.14196 (PMC10034621; doi:10.1111/1751-7915.14196)
Supplement: Supplementary file 1 — Table S1 [file MBT2-16-799-s001.docx]

**Table S1.** Antibodies used for Western blotting

| **Target** | **Provider** | **Catalogue** | **Dilution** |
| --- | --- | --- | --- |
| GPR109A | Bioss | bs-10079R | 1:10,000 |
| ACE2 | Abclonal | A4612 | 1:10,000 |
| AT1R | Proteintech | 25343-1-AP | 1:10,000 |
| AT2R | Bioss | bs-0438R | 1:10,000 |
| ANP | HUABIO | ET1705-75 | 1:10,000 |
| BNP | Abcam | ab239510 | 1:10,000 |
| β-MHC | Abclonal | A7564 | 1:10,000 |
| α-SMA | Proteintech | 14395-1-AP | 1:10,000 |
| GLP-1R | HUABIO | ER1909-68 | 1:10,000 |
| AMPK | Abclonal | A4344 | 1:10,000 |
| p-AMPK | Boster | P00994 | 1:10,000 |
| mTOR | Proteintech | 66888-1-Ig | 1:10,000 |
| p-mTOR | Affinity | BM4840 | 1:10,000 |
| p70S6K | Proteintech | 14485-1-AP | 1:10,000 |
| p-p70S6K | Affinity | AF3227 | 1:10,000 |
| 4EBP1 | Proteintech | 60246-1-Ig | 1:10,000 |
| p-4EBP1 | Affinity | AF7376 | 1:10,000 |
| β-actin | CST | 3700s | 1:10,000 |
| Goat-anti mice IgG^HRP^ | Proteintech | SA00001-1 | 1:5,000 |
| Goat-anti rabbit IgG^HRP^ | Proteintech | SA00001-2 | 1:5,000 |
